# Hybrid ICA—Regression: Automatic Identification and Removal of Ocular Artifacts from Electroencephalographic Signals

**DOI:** 10.3389/fnhum.2016.00193

**Published:** 2016-05-03

**Authors:** Malik M. Naeem Mannan, Myung Y. Jeong, Muhammad A. Kamran

**Affiliations:** Department of Cogno-Mechatronics Engineering, Pusan National UniversityBusan, South Korea

**Keywords:** electroencephalography, electrooculography, ocular artifacts, independent component analysis, regression analysis, median absolute deviation, composite multi-scale entropy, kurtosis

## Abstract

Electroencephalography (EEG) is a portable brain-imaging technique with the advantage of high-temporal resolution that can be used to record electrical activity of the brain. However, it is difficult to analyze EEG signals due to the contamination of ocular artifacts, and which potentially results in misleading conclusions. Also, it is a proven fact that the contamination of ocular artifacts cause to reduce the classification accuracy of a brain-computer interface (BCI). It is therefore very important to remove/reduce these artifacts before the analysis of EEG signals for applications like BCI. In this paper, a hybrid framework that combines independent component analysis (ICA), regression and high-order statistics has been proposed to identify and eliminate artifactual activities from EEG data. We used simulated, experimental and standard EEG signals to evaluate and analyze the effectiveness of the proposed method. Results demonstrate that the proposed method can effectively remove ocular artifacts as well as it can preserve the neuronal signals present in EEG data. A comparison with four methods from literature namely ICA, regression analysis, wavelet-ICA (wICA), and regression-ICA (REGICA) confirms the significantly enhanced performance and effectiveness of the proposed method for removal of ocular activities from EEG, in terms of lower mean square error and mean absolute error values and higher mutual information between reconstructed and original EEG.

## Introduction

Nowadays, researchers have been using non-invasive neuro-physiological techniques to understand the functional dynamics of the brain (Jöbsis, 1977; Friston et al., 1994; Kiebel et al., 2009; Kamran et al., 2012, 2015; Hogervorst et al., 2014). Electroencephalography (EEG) is a portable and high-temporal resolution brain-imaging method that can be used for quantitative analysis of the brain's different functional states (Gwin et al., 2010; Kamran and Hong, 2013, 2014; Zeng et al., 2013; Sameni and Gouy-Pailler, 2014; Schmüser et al., 2014). However, a recorded EEG signal is highly contaminated with non-neuronal activities from different sources like eye blinking, eye movements, muscle movements, and electrocardiography (ECG) (Xia et al., 2014; Zaho et al., 2014; Mannan et al., 2016). Eye movements and blinking generate major artifacts with high magnitudes as compared to the pure neuronal activity (Berg and Scherg, 1991; Wallstrom et al., 2004; Dimigen et al., 2011). Such interferences are commonly known as ocular artifacts (Corby and Kopell, 1972; Gratton et al., 1983). Also, it has been proven that these artifacts diminish the classification accuracy of brain-computer interface (BCI) applications (Fatourechi et al., 2007). Therefore, for EEG signal analysis, a method is required that can efficiently remove ocular artifacts without distorting and losing neuronal-activity-related EEG signals.

In the past, several manual and automated methods have been developed to deal with this challenging task. One straightforward approach to the reduction of ocular artifacts is to prevent eye movements as much as possible, though requiring this and achieving it are two very different things. Another commonly employed solution is to discard those epochs of EEG data that contain ocular artifacts, but this can also cause to loss neuronal-activity-related EEG data. Alternatively, several automated methods for detection and removal/reduction of ocular artifacts have been proposed. These methods can be divided into two groups: regression-based methods (Croft and Barry, 1998a,b, 2000a,b; Wallstrom et al., 2004; Croft et al., 2005; Romero et al., 2009) and blind source separation techniques (Barbati et al., 2004; Joyce et al., 2004; Hoffmann and Falkenstein, 2008; Ghandeharion and Erfanian, 2010; Javidi et al., 2011; Winkler et al., 2011; Mahajan and Morshed, 2015).

**Figure 1:**
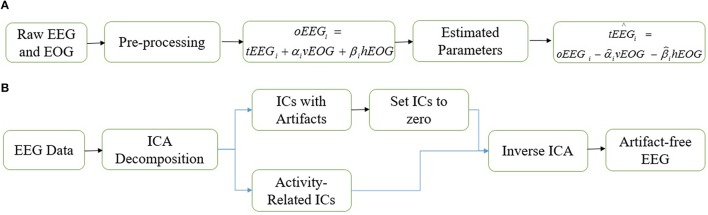
**Schematic diagrams. (A)** Regression method. **(B)** Independent component analysis.

Regression methods are based on a simple methodology entailing the subtraction of electrooculography (EOG) signals from EEG signals after estimation of the ocular artifacts propagation coefficients (He et al., 2004; Klados et al., 2011; Peng et al., 2013). Removal of ocular activities using regression method is based on an invalid assumption that there is no correlation between the neuronal activity in EEG signal and EOG signals (Sadasivan and Narayana, 1996; Jervis et al., 1998). As a result, regression analysis eliminates the neuronal activity common to both EEG and EOG from EEG signals. Regression methods are computationally simple but their outcome is highly effected by bidirectional contamination (Klados et al., 2011). The schematic diagram of a representative regression method is shown in Figure 1A.

Alternative to regression methods, blind source separation assumes that artifactual activities and cerebral activities are independent of each other. Independent component analysis (ICA) is the most commonly employed method which utilizes the assumptions of blind source separation technique (Iriarte et al., 2003; Barbati et al., 2004; Kong et al., 2013). In general, ICA decomposes multi-channel EEG signals from different sources into independent components (ICs). Recently, ICA emerged as a valuable tool for removing ocular artifacts from EEG signals because it does not experience the disadvantages that are suffered by regression analysis. Although the performance of ICA is promising, it should be employed with care (Stone, 2002). Most of the ICA based studies focused extensively on the removal of artifacts from EEG signals (Tran et al., 2004), while the effect of the method on the neuronal part of EEG signals have been neglected (Castellanos and Makarov, 2006). Additionally, the selection of artifactual components has been done by visualizing the topographic maps and time series of the ICs (Jung et al., 2000a; Iriarte et al., 2003; Makeig and Onton, 2009), thus highly depending on the expertise of the researcher. Usually this manual identification leads to divergent results (Plöchl et al., 2012). In recent years several automated methods to identify and remove artifactual ICs have been developed (Bian et al., 2006; Mammone and Morabito, 2008; Winkler et al., 2011; Plöchl et al., 2012; Kong et al., 2013). These methods proved to be effective in terms of artifact reduction and computational cost but removing artifactual ICs may cause some amount of neuronal activity loss from the EEG signal (Barbati et al., 2004; Castellanos and Makarov, 2006; Klados et al., 2011). Figure 1B demonstrates the removal of ocular artifacts using ICA.

**Figure 2:**
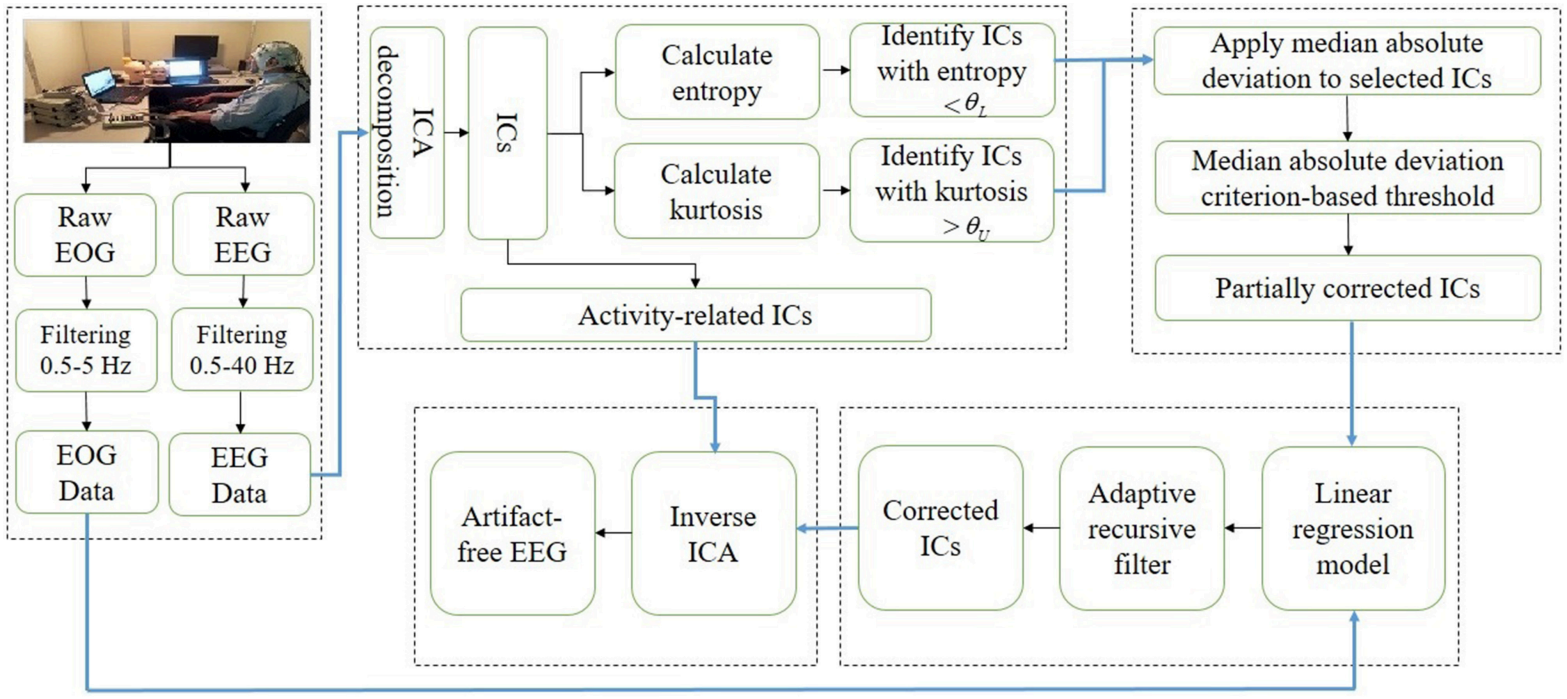
**Schematic diagram of the proposed method**.

This study presents a novel automatic artifact removal methodology for EEG signals analysis that proceeds by combining the advantageous features of ICA and regression analysis. ICA is used to decompose EEG data into different ICs, which are then separated into neuronal and ocular components by two statistical measures, namely composite multi-scale entropy and kurtosis. Then, in the proposed method's second step, high-magnitude ocular activities are removed from identified artifact-related ICs by using median absolute deviation. In the third step, the artifact-related ICs are further processed to a linear regression model in order to completely remove ocular artifacts and to recover the neuronal activities from the ICs. Finally, in the fourth step, all of the ICs (neuronal-activity-related ICs and ICs reconstructed by the proposed method) are back-projected to reconstruct artifact-free EEG signals. In this study, we assumed that artifactual activities are included in few components, therefore, the neuronal activity present in those components will be minimal. It can be further assumed that the neuronal activity present in artifactual ICs is very low as compared to that of present in EEG, which means that the common neuronal activity between artifactual ICs and EOG signal will be minimum. In this way, applying regressions to artifactual ICs causes less removal of neuronal activity. Thus, only ocular artifacts related to EOG signals are removed, while the remaining neuronal activity in the artifactual component is projected back. In this way the reconstructed EEG incorporates more neuronal information compared to the previous methods. Moreover, simulated datasets are utilized to compare and evaluate the effectiveness of the proposed method with four conventional methodologies from the literature, (1) ICA (Mognon et al., 2010), (2) least mean square based regression method, (3) wICA (Castellanos and Makarov, 2006), and (4) REGICA (Klados et al., 2011). The performance evaluation indexes employed are the mean square error and the mean absolute error in time- and frequency-domain, respectively. Additionally, mutual information is utilized to estimate the common information between reconstructed EEG signal and artifact-free EEG signal. Also, significant improvement in removing ocular activities from EEG data achieved by the proposed method relative to the conventional methods is statistically validated by using paired *t*-test. Moreover, the qualitative results on real EEG datasets further enhance the effectiveness of the proposed method. The results demonstrate that the proposed framework can be utilized as an effective method for automatic detection and removal of ocular activities from EEG data. Figure 2 shows the schematic diagram and Table 1 lists the summary of the proposed method, respectively.

**Table 1:** **Summary of the proposed method**.

**Input: Contaminated EEG data, vertical EOG, horizontal EOG**
**Output: Artifact-free EEG data**
1. Decompose contaminated EEG data using ICA to get ICs
2. Calculate composite multi scale entropy and kurtosis to identify ocular artifact related ICs
3. Apply median absolute deviation to remove high magnitude ocular artifacts
4. Filter ICs with linear regression model and extended recursive least squares
5. Artifact-free EEG data by back projecting all ICs using inverse ICA

## Materials and methods

### Materials

We used simulated, experimental, and standard EEG datasets to illustrate the effectiveness of the proposed method.

#### Simulated datasets

The primary tool to analyze the performance and effectiveness of any method is its utility for removal of ocular activities from simulated EEG datasets. For this analysis, artificially simulated EEG datasets with EOG signals were sampled from Klados et al. (2011). In this dataset, 54 artifact-free (27 in eye open session and 27 in eye closed session) EEG and EOG epochs of 30 s duration were recorded from 27 healthy participants. Nineteen EEG electrodes positioned according to the international 10–20 system (Fp1, Fp2, F3, F4, F7, F8, Fz, C3, C4, Cz, T7, T8, P7, P8, P3, P4, O1, and O2) were used for acquiring EEG signals. Electrodes with odd and even indices were referenced to the left and right mastoids, respectively. Central electrodes were referenced to the half of the sum of the left and right mastoids. In this study, we only used 12 datasets to analyze the performance and effectiveness of the proposed method. All the data were sampled at 200 Hz. The EEG and EOG data were filtered between 0.5–40 and 0.5–5 Hz, respectively.

Finally, the contaminated EEG was generated by Elbert's contamination model (Elbert et al., 1985) as

(1) $$\begin{array}{l} CEEG_{i}=PEEG_{i}+a_{i}vEOG+b_{i}hEOG, \end{array}$$

where $CEEG_{i}\in {ℜ}^{1\times N}$ and $PEEG_{i}\in {ℜ}^{1\times N}$ are the artificially contaminated and pure EEG signals, *vEOG* ∈ ℜ^1×*N*^ and *hEOG* ∈ ℜ^1×*N*^ are the vertical and horizontal EOG signals, *N* is the sample size, *a*_*i*_ ∈ ℜ and *b*_*i*_ ∈ ℜ are the contamination coefficients initialized according to Lins et al. (1993) and *i* represents the electrode.

#### Experimental datasets

Simultaneous EEG and EOG data were recorded from 11 healthy participants, all male, mean age 28. Experiment was performed in accordance with the guidelines of the Declaration of Helsinki. All participants provided written, informed consent to participation in this study. Experimental protocol was approved by the Institutional Review Board of Pusan National University. The participants were students of Pusan National University who all had reported normal or corrected-to-normal vision. The experiment was performed in a quiet room with dim lighting to prevent environmental disturbances.

Each participant was seated in an armchair at a distance of about 1 m from a 15.6″ laptop screen (Samsung, resolution 1366 × 768). The experimental protocol was as follows. At the start of the experiment the subject was instructed to close his eyes for 10 s. A sound beep was used to indicate the subject to open their eyes. Five different word cues (blink, move up, move down, move right, move left) were used in the experiment. Subjects were asked to blink their eyes or move them vertically or horizontally according to the eight visual cues that appeared for 2 s each at the center of the screen. The interval between the cues was 3 s. At the end of experiment, each subject was again asked to close his eyes for 10 s.

**Figure 3:**
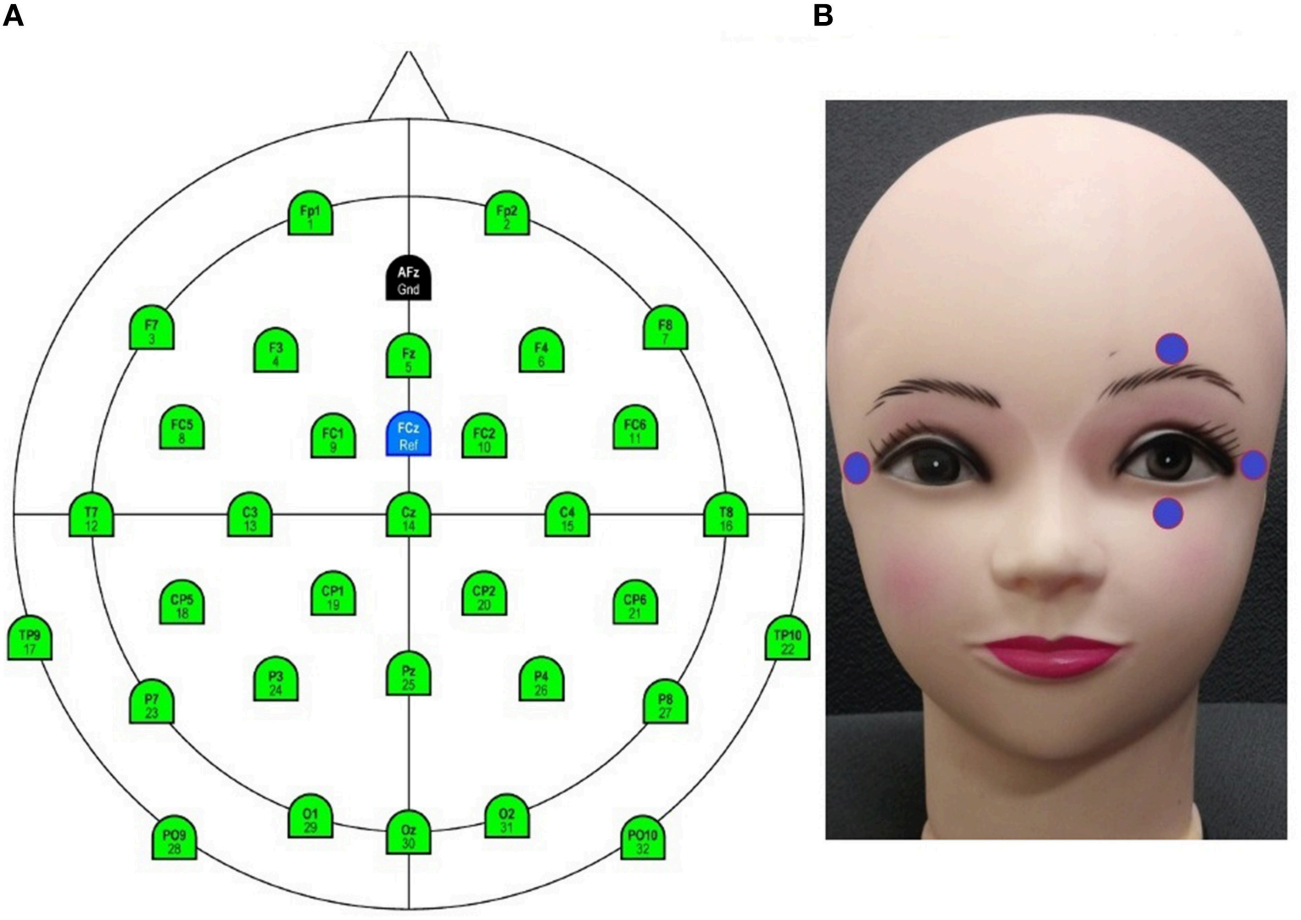
**Electrode configurations (A) Electroencephalogram (EEG). (B)** Electrooculogram (ExG).

EEG data were acquired using an ActiCap 32-channel active electrode system with a BrainAmp DC amplifier (Brain Products GmbH, Gilching, Germany). The sampling rate of the data was 250 Hz. The scalp electrodes were positioned, as shown in Figure 3A, according to the international 10–20 system. The impedance of all electrodes was reduced below 5*k*Ω. The data were band pass filtered between 0.5 and 40 Hz.

EOG data were acquired using the BrainAmp ExG system (Brain Products GmbH, Gilching, Germany). The electrode placement was as indicated in Figure 3B. The data were band pass filtered between 0.5 and 5 Hz.

#### Standard dataset

The principal evaluation measure of the effectiveness of the proposed method is its ability for removal of ocular activities from standard datasets. For this, an ocular artifacts-contaminated EEG datasets with EOG signals were sampled from the Physiobank database (Goldberger et al., 2000). In this dataset, each participant had been asked to spell a total of 20 characters from a 6 × 6 matrix speller. The signals recorded comprised 64 EEG electrodes, two earlobes referencing, and horizontal and vertical EOG for artifact removal. Twelve subjects' data were acquired with a sampling rate of 2048 Hz (see Citi et al., 2010 for details). In the preprocessing part, we removed the baseline from all of the data. The EEG and EOG data were band pass filtered between 0.5–40 and 0.5–5 Hz, respectively.

### Methods

#### Independent component analysis

ICA is a computational and statistical method used to decompose multi-channel datasets into various ICs under the following assumptions (Delorme et al., 2007):

1. The maximum number of ICs must be less than or equal to the number of electrodes used to acquire EEG data;
2. The neuronal and artifactual sources are independent of each other and linearly mixed;
3. Propagation delays from the brain sources to the electrodes are negligible.

The basic aim of ICA is to find linear projections of the data that maximizes their mutual independence. Mathematically,

(2) $$\begin{array}{l} \text{x}\left(k\right)=\text{As}\left(k\right),\;k=1,2,3,\ldots ,N \end{array}$$

where **x**(*k*) ∈ ℜ^*M*×1^ represent the recorded EEG signals, **s**(*k*) ∈ ℜ^*M*×1^ represent the corresponding ICs, **A** ∈ ℜ^*M*×*M*^ represent the full rank unknown mixing matrix, *k* represents the discrete time, and *M* represents the number of electrodes. Since the ICs contributing to EEG signals are unknown, in this study it was supposed to be equal to the number of EEG electrodes used to record signals. Given **x**(*k*), the issue is how to estimate both **A** and **s**(*k*). The ICs ${\hat{\text{s}}}_{i}\left(k\right),i=1,2,3,\ldots ,M$ can be described as

(3) $$\begin{array}{l} {\hat{\text{s}}}_{i}\left(k\right)={\text{w}}_{i}^{T}\text{x}\left(k\right),\;k=1,2,3,\ldots ,N \end{array}$$

where **w**_*i*_ is a column vector. After estimation of each **w**_*i*_, the ICs can be calculated as

(4) $$\begin{array}{l} \hat{\text{s}}\left(k\right)=\text{W}\text{x}\left(k\right),\;\text{W}\approx {\text{A}}^{-1} \end{array}$$

We employed infomax ICA method with default parameters using the *runica* function of the EEGLAB tool box (MATLAB, CA, US) (Delorme and Makeig, 2004). These parameters involved pre-sphering of the data to stop training if the weight change was < 10^−6^.

#### Regression model

The regression methods assumes that the observed EEG signal is a combination of the EOG signals and the true EEG signal, where the latter is the unobserved signal that would have been recorded without any artifact contamination. The linear model to relate the observed EEG signal to the observed EOG signal and the true EEG signal can be represented as Klados et al. (2011)

(5) $$\begin{array}{l} oEEG_{i}=tEEG_{i}+\alpha_{i}vEOG+\beta_{i}hEOG, \end{array}$$

where $oEEG_{i}\in {ℜ}^{1\times N}$ and $tEEG_{i}\in {ℜ}^{1\times N}$ are the observed and true EEG signals at the *i*th electrode respectively.

In this study, an extended recursive least squares method was utilized to approximate the parameters of the regression model. Extended recursive least squares is an adaptive tracking scheme that estimates the state of a process by employing a recursively updated regularized linear inversion routine. Extended recursive least squares offers a better tracking performance in finding optimal parameters than does the recursive least squares method (Liu et al., 2009). The extended recursive least squares method is an adaptive filter which recursively finds the coefficients that minimize a cost function relating to the input signals. The coefficients are estimated in each recursion on the basis of error in the estimation. Mathematically, it proceeds (Hansen et al., 2013) by expressing the linear regression model Equation (5) for a particular electrode *i* as

(6) $$\begin{array}{l} \varepsilon_{i}\left(k\right)=oEEG_{i}\left(k\right)-oEÊG_{i}\left(k\right), \end{array}$$

(7) $$\begin{array}{l} oEÊG_{i}\left(k\right)=X\left(k\right){\hat{\theta }}_{i}\left(k-1\right), \end{array}$$

(8) $$\begin{array}{l} {\hat{\theta }}_{i}\left(k\right)={\hat{\theta }}_{i}\left(k-1\right)+K\left(k\right)\varepsilon_{i}\left(k\right), \end{array}$$

(9) $$\begin{array}{l} K\left(k\right)=P\left(k-1\right)X\left(k\right){\left[I+X^{T}\left(k\right)P\left(k-1\right)X\left(k\right)\right]}^{-1}, \end{array}$$

(10) $$\begin{array}{rcl} P\left(k\right) & = & P\left(k-1\right)-P\left(k-1\right)X\left(k\right)X^{T}\left(k\right)P\left(k-1\right)\\ \left[I+X^{T}\left(k\right)P\left(k-1\right)X\left(k\right)\right], \end{array}$$

where ε_*i*_(*k*) is the estimation error, *oEÊG*_*i*_ is the estimation of *oEEG*_*i*_, *X*(*k*) is the matrix containing *vEOG*(*k*) and *hEOG*(*k*), ${\hat{\theta }}^{i}=\left[{\hat{\alpha }}_{i}{\hat{\beta }}_{i}\right]$ is the parameter vector, *K*(*k*) is the weighting vector for parameter updating, and *P*(*k*) is the input covariance matrix updated at each time step *k*. The initial value *P*(*k*) is chosen to be δ*I*, δ = 10.

Finally, the estimated true EEG can be calculated by subtracting the estimated EOG signals from the observed EEG signal using the formula

(11) $$\begin{array}{l} {\overset{\wedge }{\mathrm{tEEG}}}_{i}=oEEG_{i}-{\hat{\alpha }}_{i}vEOG-{\hat{\beta }}_{i}hEOG, \end{array}$$

where the indicator $\hat{.}$ represents the estimated variables.

#### Features for identification of ocular artifacts-related ICs

##### Composite multi-scale entropy

Quantifying the amount of regularity for identification of the artifactual ICs in a physiological signal using entropy has been found to be very useful. An efficient method to compute the entropy values of the physiological signals was developed (Costa et al., 2005), and has been successfully applied to extract more information on physiological signal regularity than can Shannon's or Renyi's entropy (Bosl et al., 2011). Thus, in the present study, ocular artifacts-related ICs were identified by composite multi-scale entropy (Wu et al., 2013).

The intuition that composite multi-scale entropy can detect ocular artifact related ICs lie in the fact that artifactual components have low entropy values because its pattern is more typical as compared to neuronal activity. So, composite multi-scale entropy could be used as a good statistical measure for recognition of ocular components. Mathematically, composite multi-scale entropy can be calculated as below:

1. Let *u*_*i*_ be the *i*th IC, the *l*th coarse-grained time series for a scale factor of τ, ${\text{z}}_{l}^{\left(\tau \right)}=\left\{z_{l,1}^{\left(\tau \right)}z_{l,2}^{\left(\tau \right)}\cdots z_{l,p}^{\left(\tau \right)}\right\}$ can be defined as
  (12) $$\begin{array}{l} z_{l,j}^{\left(\tau \right)}=\frac{1}{\tau }\overset{\tau +l-1}{\underset{i=\left(j-1\right)\tau +l}{\sum }}u_{i},\;1\leq j\leq \frac{N}{\tau },1\leq l\leq \tau \end{array}$$
2. In the composite multi scale entropy algorithm, at a scale factor of τ, the sample entropies (SampEns) of all coarse-grained time series are calculated and the composite multi scale entropy value is defined as the mean of τ entropy values. That is
  (13) $$\begin{array}{l} CMSE\left(\text{u},\tau ,m,r\right)=\frac{1}{\tau }\overset{\tau }{\underset{l=1}{\sum }}SampEn\left({\text{z}}_{l}^{\left(\tau \right)},m,r\right). \end{array}$$

where *CMSE* defines composite multi scale entropy. In this paper, the composite multi scale entropy was calculated from τ = 1 to 20, and the sample entropy of each coarse-grained IC was calculated with *m* = 2 and *r* = 0.15σ, where σ is the standard deviation of the IC (Costa et al., 2005; Wu et al., 2013).

##### Kurtosis

Kurtosis is a statistical measure of peakedness of distributions in random variables. The global kurtosis coefficient of the *i*th IC can be computed (Barbati et al., 2004) as

(14) $$\begin{array}{l} K\left(i\right)=m_{4}\left(i\right)-3m_{2}^{2}\left(i\right), \end{array}$$

(15) $$\begin{array}{l} m_{n}\left(i\right)=E\left\{{\left(x\left(i\right)-m_{1}\left(i\right)\right)}^{n}\right\}, \end{array}$$

where *m*_*n*_ is the *n*th central moment, *m*_1_ is the mean, and *E* is the expectation operator. Ocular artifacts-related activities typically have a peaked distribution and a high kurtosis value in EEG recordings. So, kurtosis can be used as a good measure to detect ocular artifacts-related ICs. In this study, an inbuilt function of Matlab, *kurt.m*, was utilized to calculate the kurtosis values of all ICs.

#### Procedure for identification of ocular components

After ICA decomposition of EEG signals, composite multi-scale entropy and kurtosis are calculated for all ICs. As ocular artifacts are notable outliers with high-magnitudes, they can be identified using reasonable threshold for entropy and kurtosis. Ocular artifacts-related ICs are identified using a two-tailed *t*-test with a 95% confidence interval. As explained earlier, the expected values of entropy for artifactual ICs is low, therefore the 95% convergence interval is used as the lower-limit threshold,

(16) $$\begin{array}{l} \theta_{L}=\bar{x}-\frac{s}{\sqrt{N}}\times t_{M-1}, \end{array}$$

where θ_*L*_ represents the lower limit for the entropy values as a threshold, $\bar{x}$ is the mean of the entropy values for all components, *s* is the standard deviation of the entropy values for all components, *M* is the total number of components, and *t*_*M*−1_ is the corresponding *t*-value. All components with entropy values below the threshold are assumed to be artifactual ICs and selected for reconstruction.

Because it is expected that the kurtosis values for artifactual ICs are high, the upper limit of the 95% convergence interval for the mean is used as the threshold:

(17) $$\begin{array}{l} \theta_{U}=\bar{x}+\frac{s}{\sqrt{N}}\times t_{M-1}, \end{array}$$

where θ_*U*_ represents the upper limit for the kurtosis value as a threshold.

#### Procedure for reconstruction of artifactual ICs

At this stage of the algorithm, artifactual ICs identified using composite multi-scale entropy and kurtosis are processed for artifact removal. ICs identified as artifactual components might also contain considerable neuronal activity (Castellanos and Makarov, 2006). Also, there is a notable difference between the amplitudes of ocular and neuronal activities. Indeed, ocular artifacts are of high magnitude, and can be localized in the time domain (Castellanos and Makarov, 2006). Removing these ICs by replacing them with zero cause considerable loss of EEG data and discards results. In this study, a multi-step methodology is adopted to tackle this problem. The first step yields the removal of high-magnitude ocular activities by setting them to zero. In this way, neuronal activity present in the artifactual ICs can be retained. In this paper, we used median absolute deviation to identify and eliminate outliers from the ICs (Leys et al., 2013). Mathematically, median absolute deviation can be calculated as below:

1. Evaluate the median absolute deviation of the identified ocular activity among the identified artifactual ICs (median absolute deviation is defined as the median of the absolute deviations of each time point from the median)
  (18) $$\begin{array}{l} MAD=bM\left(\left|u_{i}\left(k\right)-M\left(u_{i}\right)\right|\right) \end{array}$$
  where *MAD* represents median absolute deviation, *M* represents the median, *M*(*u*_*i*_) represents the median of the *i*th artifactual IC, *b* is a constant;
2. If *u*_*i*_(*j*) exceeds the criteria calculated using Equation (20), it is thresholded to zero:
  (19) $$\begin{array}{l} M\left(u_{i}\right)-3\times MAD<u_{i}\left(k\right)<M\left(u_{i}\right)+3\times MAD, \end{array}$$
  (20) $$\begin{array}{l} \frac{u_{i}\left(k\right)-M\left(u_{i}\right)}{MAD}>|\;\pm \;3|. \end{array}$$

This procedure will remove only those ocular activities that can be easily visualized and are identifiable in the artifactual ICs. In the next step, the ICs are processed to remove further ocular artifacts and to compensate for the possible loss of the neuronal signal in the first step. The expected neuronal activity present in the contaminated EEG signal is much higher as compared to that of present in the artifactual component, therefore it can be supposed that these ICs contain less information similar to EOG signals. In this way, applying regression to artifactual ICs will result in less removal of useful information. A linear regression model can be used to describe the contamination of artifactual ICs according to the linear model (Equation 5). So, the model described in Equation (5) receives artifactual ICs as input and EOG signals as reference. To estimate the coefficients of the EOG signals, an adaptive filter based on the extended recursive least squares described in Equations (6–10) can be used. The artifact-free ICs are calculated by subtracting the estimated EOG for the contaminated ICs using Equation (11). Finally, all of the ICs are back-projected to reconstruct the cleaned EEG data.

## Alternative methods

In this section, the conventional methods used for comparison purpose are described. Additionally, the procedure for manual detection of artifactual ICs by experts is also described.

### Regression method

Regression method is implemented as follows

1. EOG parameters are estimated by applying least mean squares estimation.
2. Estimated *v*EOG and *h*EOG are subtracted from EEG to reconstruct artifact-free EEG.

### ADJUST based ICA

ICA method is implemented as described by Mognon et al. (2010). The step-wise implementation is described below

1. ICA decomposition of EEG signals.
2. Artifactual ICs are identified by combining stereotyped artifact-specific spatial and temporal features.
3. Features are optimized to detect eye movements and blinks.
4. Removal of artifactual ICs.
5. Inverse ICA to obtain artifact-free EEG signals.

In this paper, a plugin of EEGLAB toolbox is used to implement ADJUST.

### Wavelet-ICA

wICA is implemented as described by Castellanos and Makarov (2006).

1. EEG signals are decomposed into ICs using ICA.
2. Apply wavelet transform to ICs.
3. Threshold wavelet coefficients to remove artifacts.
4. Inverse wavelet transform to obtain artifact-free ICs.
5. wICA reconstructed EEG by inverse ICA.

### REGICA

REGICA is implemented as follows (Klados et al., 2011)

1. ICA decomposition of EEG signals.
2. Filter the ICs using stabilized recursive least squares method with EOG signal as reference.
3. Back projection of all ICs to reconstruct EEG signal.

In this paper, REGICA has been implemented as a plugin of the EEGLAB toolbox.

### Identification of the artifactual ICs by experts

In this article, a combination of kurtosis and entropy based identification criteria is developed to use for automatic detection of ocular artifact related ICs. The validation of this identification procedure is done by comparing the results with manual identification of two independent EEG experts. For this validation, topographic maps and time series of all the ICs were shown to the experts to identify ocular artifact related ICs.

## Evaluation indexes

### Mean square error and mean absolute error

In this paper, the performance and effectiveness of the proposed method and conventional methods were evaluated by utilizing the mean square error and mean absolute error criterion. Mean square error was defined (Peng et al., 2013) as

(21) $$\begin{array}{l} MSE=\overset{N}{\underset{n=1}{\sum }}\frac{{\left[EEG_{out}-EEG_{in}\right]}^{2}}{N}, \end{array}$$

where *EEG*_*out*_ is the reconstructed EEG from the proposed method and *EEG*_*in*_ is the artifact-free EEG. An effective method is supposed to remove all artifacts so that the output EEG is as close as possible to the pure EEG; thus, the mean square error between them should be as low as possible.

Mean absolute error is defined to measure the distortion across different frequency bands, delta (0.5–4 Hz), theta (4–8 Hz), alpha (8–12 Hz), beta (12–30 Hz), and gamma (30–40 Hz) (Klados et al., 2011)

(22) $$\begin{array}{l} MAE=|P_{inEEG}-P_{outEEG}|, \end{array}$$

where *P* denotes the power spectrum density (PSD). PSD is estimated using Welch method with parameters, 200 sample points as the length of window and 5 sample points overlap. The average PSD for each frequency band was calculated for all subjects.

Finally, paired *t*-test was utilized to statistically compare the MSE and MAE values for the proposed method on each simulated dataset with those of ICA, regression analysis, wICA, and REGICA.

### Mutual information

The amount of mutual information between reconstructed EEG signal by the proposed method and artifact-free EEG is calculated to analyze the ability of the proposed method in recovering the neuronal activity related EEG signal. Mathematically, it can be calculated as follows (Ghandeharion and Erfanian, 2010)

(23) $$\begin{array}{l} MI=\overset{\infty }{\underset{-\infty }{\int }}\overset{\infty }{\underset{-\infty }{\int }}f\left(a,b\right)\log\frac{f\left(a,b\right)}{f\left(a\right)f\left(b\right)}dadb, \end{array}$$

where *f*(*a, b*) represents the joint pdf and *f*(*a*) and *f*(*b*) represent the marginal pdfs. The artifact-free EEG and reconstructed EEG from the proposed method are closely related if and only if the mutual information values between them are large.

## Results

The present study proposed to use a combination of entropy and kurtosis to improve the automatic detection of artifactual ICs. The results of this automatic identification are compared with the manual identification of two EEG experts. Notably, both experts' classification of ocular artifact related ICs was identical. Additionally, the performance of the automatic identification is also compared with ADJUST based identification of ocular artifacts related ICs (Mognon et al., 2010). Furthermore, four statistical measures are calculated to verify the ability of the automatic identification procedure to detect the artifactual ICs. True Positive (ICs identified as artifact related by the method and experts), False Positive (ICs identified by the method but not by experts), True Negative (ICs identified neither by the method nor by experts), and False Negative (ICs not identified by the method but by experts) are calculated to compare the performance and effectiveness of the proposed method and ADJUST. Table 2 list the results of each measure for all subjects. Moreover, average sensitivity and average specificity are computed as below (Mahajan and Morshed, 2015):

(24) $$\begin{array}{l} Sensitivity=\frac{TP}{TP+FN}\times 100\% \end{array}$$

(25) $$\begin{array}{l} Specificity=\frac{TN}{TN+FP}\times 100\% \end{array}$$

**Table 2:** **Performance comparison of the proposed method and ADJUST for identification of ocular artifact related ICs with manual detection**.

**Parameters/Methods**	**Proposed**	**ADJUST**
True Positive (TP)	51	44
False Positive (FP)	10	13
True Negative (TN)	285	282
False Negative (FN)	6	13
Average Sensitivity	89.47%	77.19%
Average Specificity	96.61%	95.59%
Agreement Rate	95.45%	92.61%

We also computed the agreement rate between the proposed method, ADJUST and manual identification (Mahajan and Morshed, 2015):

(26) $$\begin{array}{l} Agreement\;Rate=\frac{TP+TN}{TP+TN+FP+FN} \end{array}$$

The results of this evaluation recommend that the proposed automatic identification can be utilized as a good tool to detect ocular artifact related components.

**Figure 4:**
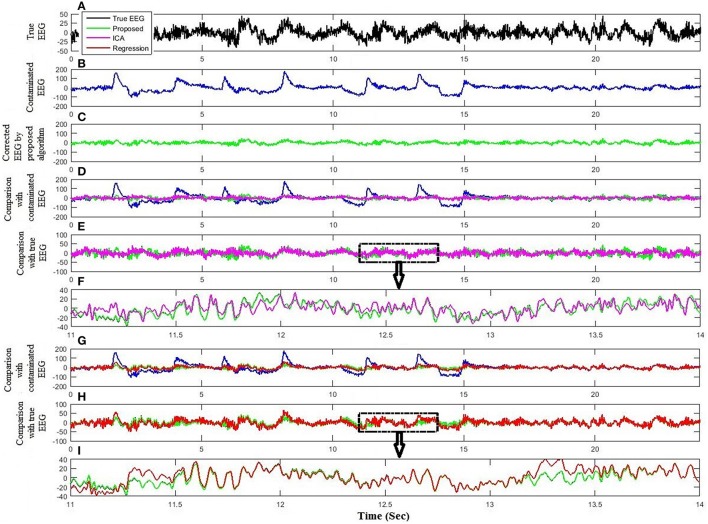
**Comparison of the proposed method with ICA and regression analysis using simulated dataset. (A)** Simulated true EEG data. **(B)** Artificially contaminated EEG data. **(C)** Reconstructed EEG by the proposed method. **(D)** Comparison of the proposed method and ICA reconstructed EEG with artificially contaminated EEG. **(E)** Comparison of the proposed method and ICA reconstructed EEG with simulated true EEG. **(F)** Partial enlargement of highlighted region. **(G)** Comparison of the proposed method and regression method reconstructed EEG with artificially contaminated EEG. **(H)** Comparison of the proposed method and regression method reconstructed EEG with simulated true EEG. **(I)** Partial enlargement of highlighted region.

**Figure 5:**
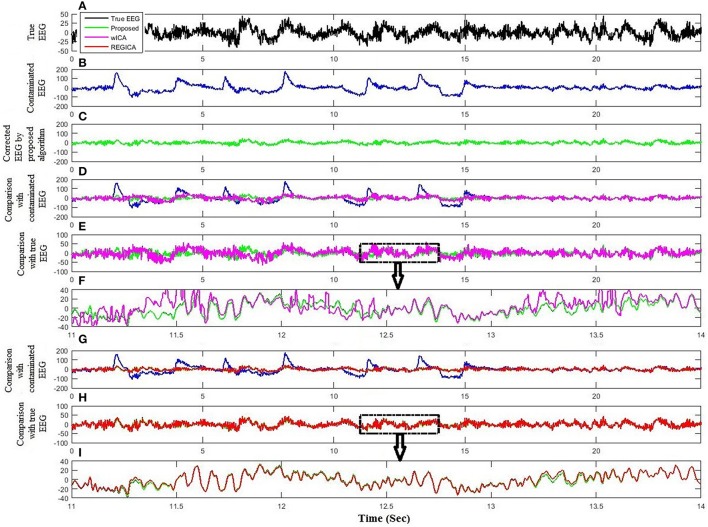
**Comparison of the proposed method with wICA and REGICA using simulated dataset. (A)** Simulated true EEG data. **(B)** Artificially contaminated EEG data. **(C)** Reconstructed EEG by the proposed method. **(D)** Comparison of the proposed method and wICA reconstructed EEG with artificially contaminated EEG. **(E)** Comparison of the proposed method and wICA reconstructed EEG with simulated true EEG. **(F)** Partial enlargement of highlighted region. **(G)** Comparison of the proposed method and REGICA reconstructed EEG with artificially contaminated EEG. **(H)** Comparison of the proposed method and REGICA reconstructed EEG with simulated true EEG. **(I)** Partial enlargement of highlighted region.

The performance and effectiveness of the proposed method is verified by utilizing 12 simulated EEG datasets. The comparison of the proposed method with ICA and regression method for the simulated dataset is shown in Figure 4. Figures 4A,B plot the simulated true EEG data and artificially contaminated EEG data, respectively. The proposed method's result of artifact removal is plotted in Figure 4C. Figure 4D shows that although both methods are successful in removing ocular artifacts, but the reconstructed EEG signals by the proposed method (green line) almost completely overlaps with the true EEG signal, whereas that from the ICA (magenta line), as depicted in Figure 4E, includes distortion. Figure 4F shows a partially enlarged region of Figure 4E highlighted with a black box, from which it can be visualized that the proposed method preserves the true EEG much better than does ICA. For the purposes of further validation, in Figure 4G, the results of the proposed method also are compared against the regression method for the simulated EEG data. As illustrated in Figure 4H, the proposed method outperformed the regression method (red line) in removing ocular activities from the simulated EEG data. From the traces in Figure 4I, it can be visualized that the regression methods tracks, to a certain extent, the EEG data in the non-artifactual zone, but still causes distortion in EEG data. Furthermore, the performance of the proposed method is assessed against wICA and REGICA in Figure 5. Figure 5F shows that wICA, although performing better than ICA, still leaves distortions as compared to the proposed method. In contrast to wICA, REGICA gives a better approximation of the artifact-free EEG signal (Figures 5G–I), but the proposed method also outperforms it (Table 3). Figure 5H also presents that both proposed method and REGICA are close enough, but Figure 5I discloses that, qualitatively, proposed method gives a closer approximation of the artifact-free EEG signal. Also, it can be difficult to distinguish true EEG (black line) in Figures 4F,I, 5F,I due to the reason that the reconstructed EEG obtained by the proposed method overlaps the true EEG. Table 3 list the average mean square error and mean absolute error values in different frequency bands for all methods for 12 simulated datasets. The results of this analysis verify the effectiveness of the proposed method over conventional methods. Paired *t*-test with 11 degrees of freedom, revealed the statistically significant difference between the proposed method and conventional methods except for delta (*p* < 0.055) and alpha (*p* < 0.028) bands in REGICA. Finally, the mutual information metric is utilized and calculated to enhance the applicability of the proposed method as compared to the previous methods. Table 4 list the average values of mutual information for all electrodes using 12 simulated datasets. It can be seen that the mutual information values of the proposed method are higher than the mutual information values of the conventional methods. In other words, the proposed method can preserve more useful information as compared to the other methods.

**Table 3:** **Average mean square error and mean absolute error values results in different frequency bands for the proposed method against conventional methods using simulated EEG datasets**.

**Bands/Method**	**Proposed**	**ICA**	***t*-test**	**Regression**	***t*-test**	**wICA**	***t*-test**	**REGICA**	***t*-test**
MSE	2.0459 ± 2.6692	14.7990 ± 21.7546	^***^	9.1388 ± 16.8010	^***^	9.5063 ± 18.5083	^***^	5.0092 ± 8.6684	^***^
Delta	0.1087 ± 0.0458	2.2660 ± 1.1004	^***^	0.3745 ± 0.2162	^***^	1.7058 ± 1.0042	^***^	0.1884 ± 0.1411	^*^
Theta	0.0293 ± 0.0128	1.0257 ± 0.3873	^***^	0.0962 ± 0.0586	^***^	0.8975 ± 0.3943	^***^	0.0453 ± 0.0221	^**^
Alpha	0.0028 ± 0.0011	0.8059 ± 0.1905	^***^	0.0054 ± 0.0027	^***^	0.9423 ± 0.7117	^***^	0.0043 ± 0.0026	^*^
Beta	0.0022 ± 0.0009	0.9501 ± 0.2670	^***^	0.0042 ± 0.0018	^***^	0.8893 ± 0.2801	^***^	0.0031 ± 0.0013	^**^
Gamma	0.0024 ± 0.0014	1.6669 ± 0.8391	^***^	0.0044 ± 0.0025	^***^	1.3765 ± 0.6174	^***^	0.0034 ± 0.0016	^**^

*MSE: mean square error*.

* *p-value is smaller than 0.06*.

** *p-value is smaller than 0.01*.

*** *p-value is smaller than 0.001*.

**Table 4:** **Average mutual information values results for the proposed method against conventional methods for all electrodes using simulated EEG data**.

**Electrode location**	**Proposed**	**ICA**	**Regression**	**wICA**	**REGICA**
Fp1	1.6398	0.9039	0.2651	1.4421	0.2259
Fp2	1.5366	0.9988	0.2342	1.3944	0.2690
F3	1.9059	1.4578	0.7306	1.8121	0.6418
F4	1.8062	1.4285	0.5596	1.7177	0.6026
F7	1.9909	1.8399	1.0204	1.8886	1.0216
F8	1.8728	1.7351	0.8462	1.7651	0.9651
Fz	1.9224	1.9765	1.0893	1.8303	1.2459
C3	1.9617	1.9696	0.8580	1.8864	1.2405
C4	1.9550	2.0387	1.1039	1.9239	1.3584
Cz	1.9929	2.0350	0.9358	1.9143	1.3500
T7	1.7554	1.2134	0.4181	1.6996	0.3862
T8	1.7768	1.2044	0.3557	1.5369	0.3581
P7	1.9318	1.5764	0.6672	1.8447	0.6997
P8	1.7724	1.5598	0.4813	1.6181	0.6670
P3	1.9143	1.7891	0.9618	1.8756	1.1355
P4	1.8039	1.8235	0.7050	1.7417	1.1603
Pz	1.8732	1.3261	0.6534	1.7142	0.5122
O1	1.9088	1.7704	0.9169	1.7895	0.9032
O2	1.9677	2.0576	1.1030	1.8811	1.2322

**Figure 6:**
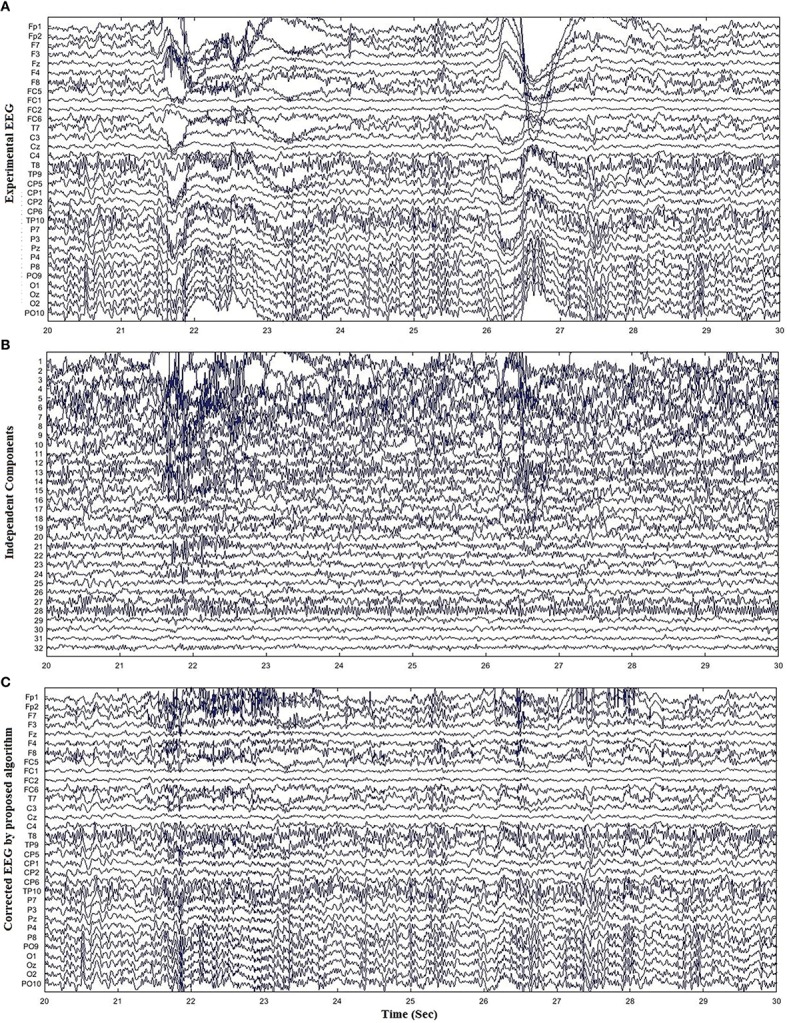
**Results for removal of ocular artifacts using experimental dataset. (A)** Experimental EEG data for one subject. **(B)** ICs obtained from ICA decomposition of EEG data. **(C)** Reconstructed EEG data by the proposed method.

**Figure 7:**
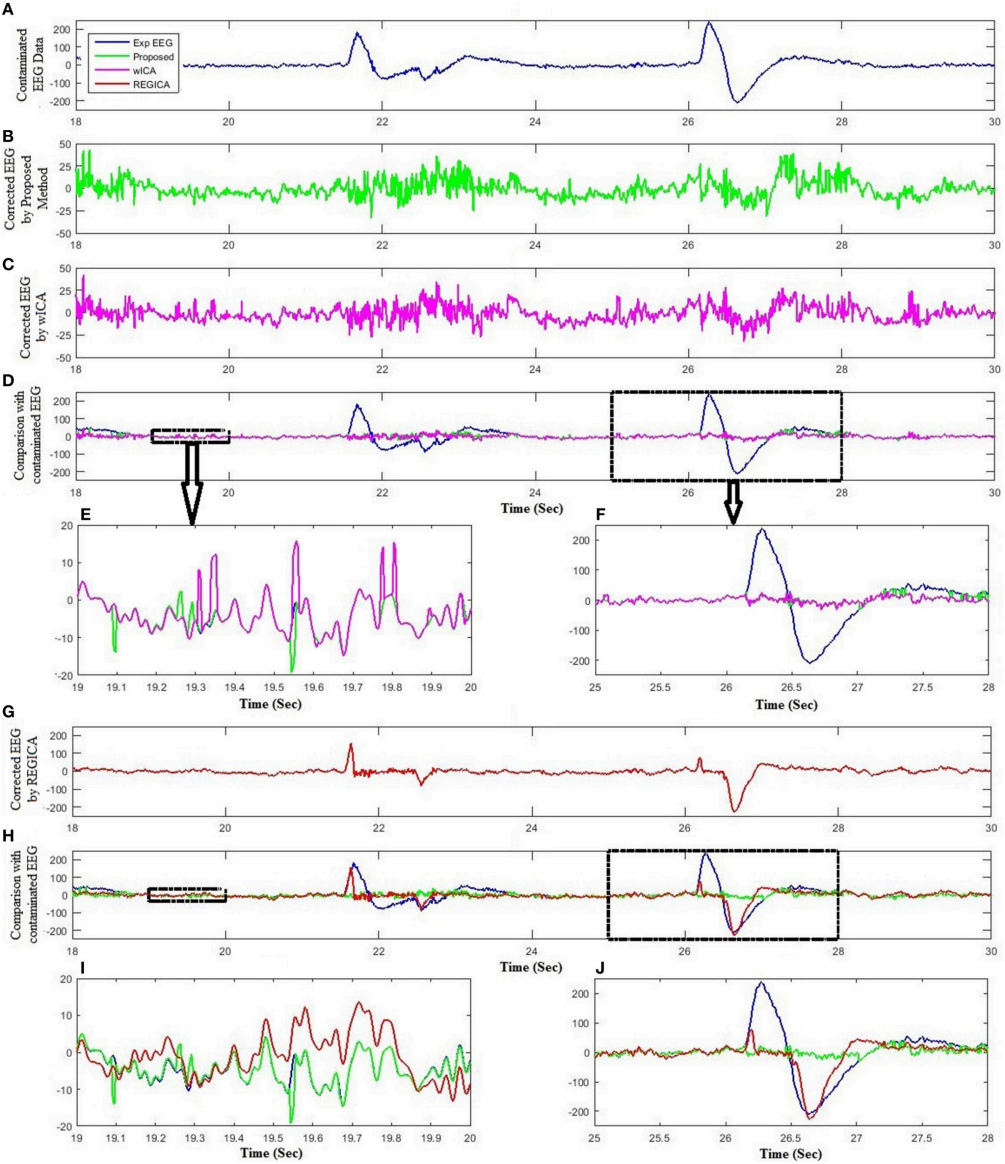
**Comparison of the proposed method with wICA and REGICA using experimental dataset. (A)** Experimental EEG data. **(B)** Reconstructed EEG by the proposed method. **(C)** Reconstructed EEG by wICA. **(D)** Comparison of the proposed method and wICA reconstructed EEG with experimental EEG. **(E)** Partial enlargement of non-artifactual region. **(F)** Partial enlargement of artifactual region. **(G)** Reconstructed EEG by REGICA. **(H)** Comparison of the proposed method and REGICA reconstructed EEG with experimental EEG. **(I)** Partial enlargement of non-artifactual region. **(J)** Partial enlargement of artifactual region.

Unlike the simulated EEG datasets, there is no pure EEG for experimental and standard EEG signals, thus only qualitative results are presented to enhance the effectiveness of the proposed method. For further verification, the proposed method was assessed for 11 experimental datasets. Figure 6 shows the artifact removal results for one subject after the application of the proposed method. Figures 6A,B display the recorded EEG signals and the corresponding ICs, respectively. Figure 6C show the reconstructed EEG obtained from the proposed method. It can be visualized that the proposed method successfully removes the ocular artifacts. Figure 7 shows the comparison of the proposed method, wICA and REGICA for removing artifacts for one subject at Fp1. Figure 7A displays the artifactual segment of the EEG signal; Figures 7B,C show the reconstructed EEG obtained after the artifact removal by the proposed method and wICA, respectively; Figure 7D shows the comparison of the contaminated EEG signal with the reconstructed EEG obtained by the proposed method and wICA. Figures 7E,F show the uncontaminated region and contaminated region of the EEG signal highlighted in Figure 7D, respectively. Figure 7E shows that the proposed method can preserve more neuronal information as compared to wICA. Figure 7F illustrates the improved performance and effectiveness of the proposed method in terms of removing artifactual activities as compared to wICA. Figure 7G shows the reconstructed EEG by REGICA; meanwhile, the comparison of the proposed framework against REGICA verify the better performance of the proposed method (Figures 7H–J).

**Figure 8:**
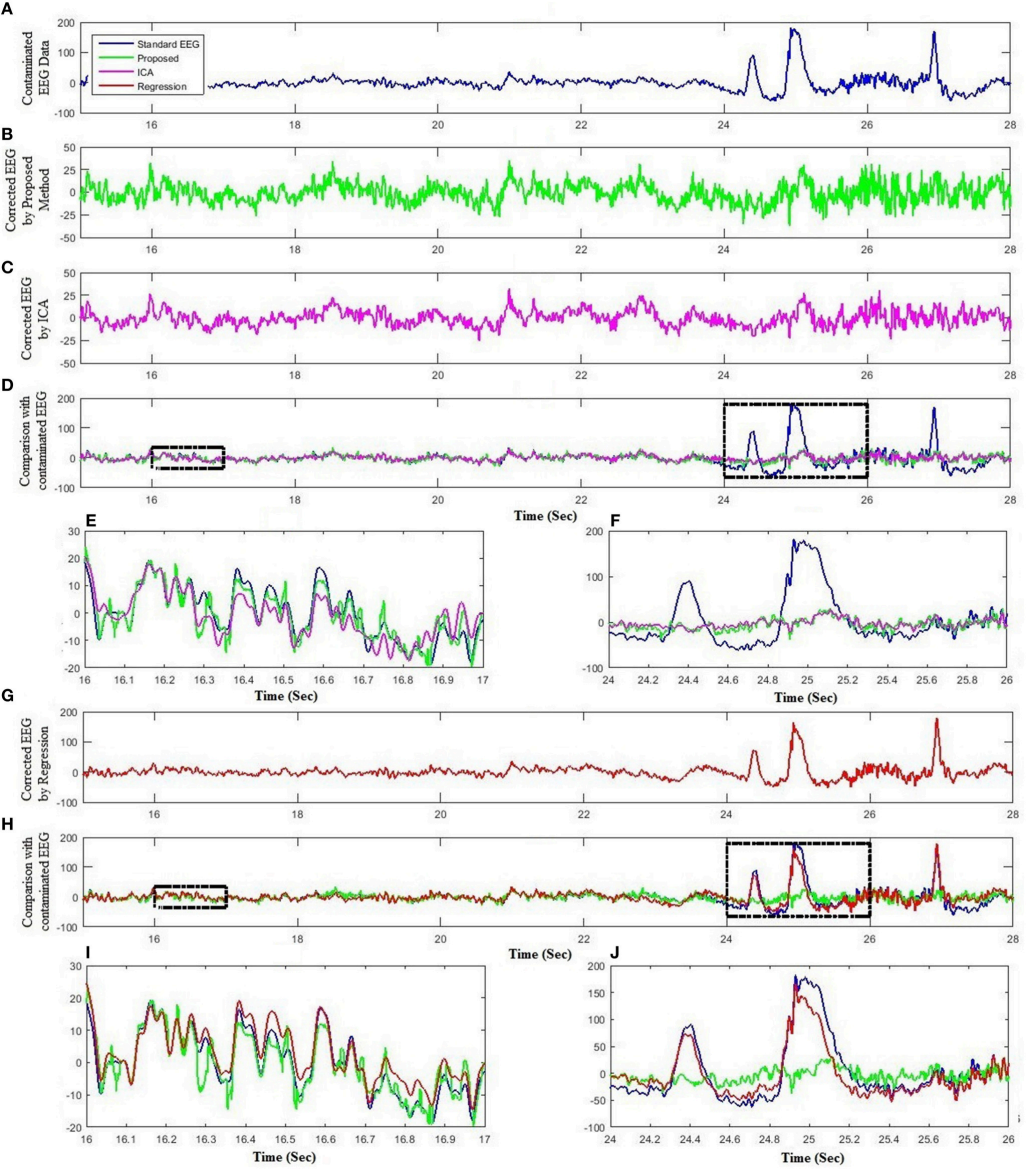
**Comparison of the proposed method with ICA and regression using standard dataset. (A)** Standard EEG data. **(B)** Reconstructed EEG by the proposed method. **(C)** Reconstructed EEG by ICA. **(D)** Comparison of the proposed method and ICA reconstructed EEG with experimental EEG. **(E)** Partial enlargement of non-artifactual region. **(F)** Partial enlargement of artifactual region. **(G)** Reconstructed EEG by regression method. **(H)** Comparison of the proposed method and regression method reconstructed EEG with experimental EEG. **(I)** Partial enlargement of non-artifactual region. **(J)** Partial enlargement of artifactual region.

Lastly, standard EEG signals are utilized to test the effectiveness of the proposed method in removing artifactual activities from contaminated EEG data. Figure 8 compares the proposed method with ICA and regression analysis for one subject at Fp1. Figures 8A–C show the segment of standard EEG signal, reconstructed EEG by the proposed method and ICA, respectively. Although both methods were successful in removing ocular artifacts, but the comparison of the reconstructed EEG by the proposed method and ICA with contaminated EEG demonstrates that the proposed method performed better in keeping the useful EEG signals (Figures 8D–F). Further, an assessment of the proposed method with the regression analysis is provided in Figures 8G,H. Similarly to the simulated and experimental study results, the proposed method demonstrates improved performance over the regression method (Figures 8I,J).

## Discussion

It is very essential to remove non-neuronal activities such as ocular artifacts from EEG signals before its analysis for applications like BCI. In this paper, a combined ICA and regression-based method is developed for automatic identification and removal/reduction of ocular artifacts from EEG data. The performance and effectiveness of the proposed method is evaluated against conventional methods to show the significant improvements in the results by utilizing simulated, experimental and standard EEG datasets along with mean square error, mean absolute error and mutual information as quantification metrics. The underlying true EEG signal in artificially contaminated EEG data is known, therefore such artificially contaminated EEG data is used as a primary tool to evaluate the performance of each artifact rejection method. The EEG data recorded in eye close session is utilized to simulate contaminated EEG. However, EEG data in eye-close session may also contain low frequency eye movements. But, the signals in eye-closed session are preferred because it seems to contain minimal artifacts. Alternative to this was acquiring EEG data in an eye open session. But human eyes produce much higher amplitude signals in light as compared to darkness (Elbert et al., 1985). In this sense, EEG signal in an eye-close session is preferred. However, EOG signals were acquired in an eye-open session with different eye movements. The recorded EOG signal was filtered between 0.5 and 5 Hz (Lins et al., 1993). In literature some studies used the limit of 7.5 Hz low pass filter (Romero et al., 2009), but there is still no evidence on the optimal low pass frequency of EOG signals. Also, in this study statistical analysis is utilized to validate the significant improvement in the performance and effectiveness of the proposed method with respect to conventional techniques.

Urigüen and Garcia-Zapirain (2015) suggested that the combination of multiple artifact removal methods can be developed to efficiently remove artifacts from the recorded EEG signals. Castellanos and Makarov (2006) were the first to develop a method which combined ICA and wavelet transform to recover the leaked-neuronal activity from artifactual components. Later on Klados et al. (2011) extended their idea and proposed a regression based method to remove ocular artifacts from ICs. These methods performed better than previous methods in terms of removing artifacts from the signals but there is no criteria to detect artifactual ICs and hence, require extra computational efforts to process all ICs which is not plausible for BCI applications. Moreover, application of these methods to all ICs may cause to loss neuronal activity related signals and may, therefore, result in distortion to EEG signals. Unlike to the conventional methods, the framework proposed in this study automatically identify and filter only the artifactual ICs. Therefore, the proposed method can be used to preserve more useful information related to neuronal activity with less computational cost. Results confirm our hypothesis that only artifact related ICs should be processed instead of processing all components (Figures 5, 7, Tables 3, 4).

The performance and effectiveness of the proposed framework is evaluated and assessed against four conventional methodologies from the literature, namely ICA (Mognon et al., 2010), least mean square based regression method, wICA (Castellanos and Makarov, 2006), and REGICA (Klados et al., 2011). In the time domain analysis, we used mean square error as a performance metric to analyze the ability of each method in both removing artifactual activities as well as calculating the amount of distortion produced in the EEG signal. Efficient removal of ocular activities and preservation of neuronal signal suggest that the proposed method performed better as compared to ICA, regression, wICA, and REGICA methods (Table 3). In addition, mean absolute error is used as a quantification metric to evaluate the effectiveness of the proposed method in frequency-domain. Results show that the proposed method produce less distortion in different frequency bands as compared to conventional methods (Table 3). Paired *t*-test also revealed that the difference between the proposed method and conventional methods is highly statistically significant (*p* < 0.001) except for delta (*p* < 0.055) and alpha (*p* < 0.028) bands of REGICA. Finally, we adopted mutual information metric to compute that how much information reconstructed EEG by different methods shares with the artifact-free EEG signals (Table 4). The average mutual information for the proposed method (1.8573) against ICA (0.7319), regression (1.6160), wICA (0.8409), and REGICA (1.7514) demonstrate better performance of the proposed method. Additionally, the performance and effectiveness of the proposed method is also analyzed using real EEG datasets (Experimental and standard EEG signals). Since there is no ground truth “pure” EEG, we have only presented qualitative results for real EEG datasets. Qualitatively, results on real EEG datasets show better performance of the proposed method as compared to the conventional methods (Figures 6–8).

Although the proposed method showed improved performance for removing ocular activities from EEG signals as compared to the conventional techniques, it also has some drawbacks/limitations. One obvious drawback of the proposed method is that it always requires EOG signals to remove ocular activities from EEG signals whereas all the ICA based methods have the ability to remove artifactual components without the need of EOG signals. However, the removal of artifactual components based on ICA cause distortion in the neuronal signal, therefore it is preferable to use EOG signals to remove ocular artifacts. Furthermore, in the present study only spontaneous EEG is considered rather than event related potentials (ERPs). In literature, ERP paradigms have been used to evaluate the performance of artifact rejection methods (Gratton et al., 1983; Jung et al., 2000b; Plöchl et al., 2012). They enhance the effectiveness of their methods by claiming that their method can keep ERPs evoked by visual stimuli. To this extent, it is our future plan to examine whether the proposed method can achieve better results in terms of preserving ERP contributions over previous methods. Besides, in the present study the proposed method only deals with ocular activities and removal/reduction of other types of artifactual activities is yet to be examined in immediate future work. One possible extension of the proposed method is to include features that can be used to identify muscle artifacts (Barbati et al., 2004). Another essential extension is to use features based on the correlation with ECG signal (Joyce et al., 2004).

## Conclusions

The optimal performance of a BCI depends on the effective removal/reduction of ocular artifacts from EEG recordings. In this paper, we proposed a novel method for automatic identification and removal/reduction of ocular artifacts from EEG signals by combined ICA, regression and high-order statistics. The performance and effectiveness of the proposed framework was illustrated using simulated and real EEG datasets. Results show that the proposed method can effectively remove ocular artifacts as well as it can preserve the neuronal activity related EEG signals. The results demonstrate, additionally, that the proposed method outperforms the conventional techniques.

## Author contributions

MN has conducted all the experiments and carried out the data processing. MJ has suggested the theoretical aspects of the current study and supervised all the process from the beginning. MK has examined the data and participated in revising the manuscript. All the authors have approved the final manuscript.

### Conflict of interest statement

The authors declare that the research was conducted in the absence of any commercial or financial relationships that could be construed as a potential conflict of interest.
